# Unraveling the safety of adjuvant radiotherapy in prostate cancer: impact of older age and hypofractionated regimens on acute and late toxicity - a multicenter comprehensive analysis

**DOI:** 10.3389/fonc.2023.1281432

**Published:** 2023-12-13

**Authors:** Milly Buwenge, Gabriella Macchia, Letizia Cavallini, Annalisa Cortesi, Claudio Malizia, Lorenzo Bianchi, Maria Ntreta, Alessandra Arcelli, Ilaria Capocaccia, Elena Natoli, Savino Cilla, Francesco Cellini, Luca Tagliaferri, Lidia Strigari, Silvia Cammelli, Riccardo Schiavina, Eugenio Brunocilla, Alessio Giuseppe Morganti, Francesco Deodato

**Affiliations:** ^1^ Department of Medical and Surgical Sciences (DIMEC), Alma Mater Studiorum University of Bologna, Bologna, Italy; ^2^ Radiation Oncology Unit, Gemelli Molise Hospital - Università Cattolica del Sacro Cuore, Campobasso, Italy; ^3^ Radiation Oncology, IRCCS Azienda Ospedaliero-Universitaria di Bologna, Bologna, Italy; ^4^ Radiotherapy Unit, IRCCS Istituto Romagnolo per lo Studio dei Tumori (IRST) “Dino Amadori”, Meldola, Italy; ^5^ Nuclear Medicine, IRCCS Azienda Ospedaliero-Universitaria di Bologna, Bologna, Italy; ^6^ Division of Urology, IRCCS Azienda Ospedaliero-Universitaria di Bologna, Bologna, Italy; ^7^ Medical Physics Unit, Gemelli Molise Hospital, Campobasso, Italy; ^8^ Fondazione Policlinico Universitario “A. Gemelli” IRCCS, Dipartimento di Diagnostica per Immagini, Radioterapia Oncologica ed Ematologia, Rome, Italy; ^9^ Department of Medical Physics, IRCCS Azienda Ospedaliero-Universitaria di Bologna, Bologna, Italy; ^10^ Istituto di Radiologia, Università Cattolica del Sacro Cuore, Roma, Italy

**Keywords:** prostate neoplasms, observational study, toxicity, predictive factors, radiotherapy, adjuvant therapy

## Abstract

**Background:**

The objective of this study was to assess the impact of age and other patient and treatment characteristics on toxicity in prostate cancer patients receiving adjuvant radiotherapy (RT).

**Materials and methods:**

This observational study (ICAROS-1) evaluated both acute (RTOG) and late (RTOG/EORTC) toxicity. Patient- (age; Charlson’s comorbidity index) and treatment-related characteristics (nodal irradiation; previous TURP; use, type, and duration of ADT, RT fractionation and technique, image-guidance systems, EQD2 delivered to the prostate bed and pelvic nodes) were recorded and analyzed.

**Results:**

A total of 381 patients were enrolled. The median EQD2 to the prostate bed (α/β=1.5) was 71.4 Gy. The majority of patients (75.4%) were treated with intensity-modulated radiation therapy (IMRT) or volumetric-modulated arc therapy (VMAT). Acute G3 gastrointestinal (GI) and genitourinary (GU) toxicity rates were 0.5% and 1.3%, respectively. No patients experienced >G3 acute toxicity. The multivariable analysis of acute toxicity (binomial logistic regression) showed a statistically significant association between older age (> 65) and decreased odds of G≥2 GI acute toxicity (OR: 0.569; 95%CI: 0.329-0.973; p: 0.040) and decreased odds of G≥2 GU acute toxicity (OR: 0.956; 95%CI: 0.918-0.996; p: 0.031). The 5-year late toxicity-free survival rates for G≥3 GI and GU toxicity were 98.1% and 94.5%, respectively. The only significant correlation found (Cox’s regression model) was a reduced risk of late GI toxicity in patients undergoing hypofractionation (HR: 0.38; 95% CI: 0.18-0.78; p: 0.008).

**Conclusions:**

The unexpected results of this analysis could be explained by a “response shift bias” concerning the protective effect of older age and by treatment in later periods (using IMRT/VMAT) concerning the favorable effect of hypofractionation. However, overall, the study suggests that age should not be a reason to avoid adjuvant RT and that the latter is well-tolerated even with moderately hypofractionated regimens.

## Introduction

Prostate cancer (PCa) is a significant health concern, ranking second in terms of incidence and fifth in terms of mortality among male populations (1). Radical prostatectomy (RP) is a commonly employed treatment option for PCa. However, the five-year biochemical relapse-free survival (bRFS) rate after RP is approximately 50% of patients with high-risk features at pathological evaluation (2–4).

Postoperative radiotherapy (RT) has been investigated as an adjunctive treatment following RP, and the results of four randomized studies (2–5) have demonstrated improved bRFS rates (around 25% at five years) compared to RP alone. Moreover, one of these studies has shown a significantly reduced risk of metastasis and improved overall survival (OS) with postoperative RT (6).

Consequently, international guidelines, such as those from the European Association of Urology^1^ (EAU 2022) and the National Comprehensive Cancer Network^2^ (NCCN 2022), recommend postoperative RT as an adjuvant therapy for selected PCa patients. Specifically, EAU guidelines recommend adjuvant RT for high-risk patients (pN0) with at least two of the following high-risk features: International Society of Urological Pathology (ISUP) grade group 4–5, pT3 stage, and positive surgical margins.

Nevertheless, recent randomized trials (7–9) and a meta-analysis (10) have demonstrated that early salvage RT can achieve biochemical and clinical outcomes comparable to those of adjuvant RT, while significantly reducing the number of patients requiring pelvic RT and improving overall treatment tolerability. These findings highlight the importance of careful patient selection for adjuvant RT, considering the cost/benefit ratio.

In this regard, it is crucial to consider both factors that predict greater benefit from adjuvant RT, such as seminal vesicle involvement (11) and positive surgical margins (12) as well as factors that indicate a higher risk of side effects. However, the available evidence on the latter topic is limited and often derived from small studies that have analyzed only specific patient and/or treatment characteristics (13–17).

Therefore, the aim of this study is to analyze multiple patient- and treatment-related factors in a large multicenter series of PCa patients who underwent adjuvant RT, with the goal of identifying predictors of increased toxicity, and in particular to evaluate whether older age is associated with a greater risk of radiation-induced side effects.

## Material and methods

### Study design and endpoints

This sub-analysis is part of a multicenter observational study (311/2019/Oss/AOUBo, ICAROS-1 study) focusing specifically on patients with PCa who underwent postoperative adjuvant RT. The study endpoints encompass both acute and late gastrointestinal (GI) and genitourinary (GU) toxicities.

### Inclusion criteria

The inclusion criteria were as follows: 1) patients diagnosed with PCa who underwent RP with negative or microscopically positive margins (R0-1) and no distant metastases, and 2) RT delivered using external beam techniques with photon beams. Exclusion criteria were: 1) presence of macroscopic (R2) residual disease after RP, 2) postoperative PSA level exceeding 0.2 ng/ml, and 3) postoperative RT delivered more than one year after RP.

### Evaluated parameters

The recorded and evaluated patient-related characteristics included age and Charlson’s comorbidity index. Age was analyzed both as a continuous variable and as a dichotomous variable using a cut-off at the median value. The analyzed treatment characteristics encompassed the delivery of prophylactic lymph node irradiation (PNI), previous transurethral resection of the prostate (TURP), use and type of adjuvant androgen deprivation therapy (ADT) (LH-RH analogues or high-dose bicalutamide) and its duration, RT fractionation and technique (including the type of image-guidance systems employed), as well as the Equivalent Dose in 2 Gy per fraction (EQD2) delivered to the prostate bed and pelvic lymph nodes. Acute toxicity was monitored with weekly visits during treatment and with a follow-up visit 2 months after the end of treatment. Late toxicity was evaluated with a first follow-up visit 6 months after the end of treatment and then with further visits every 6 months up to 24 months after treatment, followed by annual assessments up to 10 years. Gastrointestinal toxicity was evaluated by patient interviews and proctoscopy, if necessary. Genitourinary toxicity was assessed through patient interviews and urine analysis during follow-up.

### Statistical analysis

Statistical computations were performed using IBM SPSS Version 22.0 software package (IBM Corp, Armonk, NY, USA). A p-value less than 0.05 was considered statistically significant. Acute toxicity was evaluated using the RTOG scale, while late toxicity was assessed using the RTOG/EORTC scale (18). The chi-squared test with Yates’ continuity correction and Fisher’s exact test were employed in univariate logistic regression to examine the correlation between the analyzed variables and acute toxicity. Additionally, a binomial logistic stepwise regression was used to estimate the likelihood of acute toxicity based on the aforementioned variables. Late toxicity-free survival estimates were calculated using the Kaplan-Meier product-limit method (19) and compared using the log-rank test (20). Variables with a p-value less than 0.05 or showing a trend (p < 0.1) in the univariate analysis were included in a multivariate Cox regression model (21).

### Ethical considerations

The study received approval from the local institutional review board, and participation in the analysis was limited to patients who provided written informed consent.

## Results

### Patients, tumors, and treatment characteristics

A total of 381 patients were included in this analysis, with a median age of 65 years (range: 43-79 years). **Table 1** presents the patients, tumor, and treatment characteristics. The median delivered EQD2 to the prostate bed, calculated using α/β ratios of 1.5 Gy, 3 Gy, and 10 Gy, was 71.4 (range: 66.2-78.0), 68.7 (range: 67.0-78.0), and 68.2 (range: 65.1-78.0), respectively. Among the patients, 127 (33.3%) were treated with standard fractionation, while 254 (66.7%) received a hypofractionated regimen. EQD2_α/β=3_ was significantly higher in patients treated with hypofractionated regimens compared to standard fractionation protocols (mean: 71.3 Gy versus 69.7 Gy; p<0.001). RT was delivered using either 3D-conformal RT (94 patients, 24.7%) or modulated techniques such as intensity modulated arc therapy (IMRT) or volumetric modulated arc therapy (VMAT) (287 patients, 75.3%). The dose to the prostatic bed ranged between 65 and 78 Gy (median: 66 Gy). Moreover, of 254 patients treated with hypofractionation, the dose per fraction was 2.2 Gy in 100 patients, 2.5 Gy in 142 patients, and 2.6 Gy in 12 patients. Furthermore, of 127 patients treated with standard fractionation, the dose per fraction was 1.8 Gy in 42 patients and 2 Gy in 85 patients. Additionally, out of 254 patients treated with hypofractionation, 239 (94.1%) were treated with IMRT/VMAT and 15 (5.9%) with 3D-CRT. Finally, out of 297 patients receiving nodal irradiation, 250 (84.2%) were treated with IMRT/VMAT, while 47 (15.8%) were treated with 3D-CRT. Daily on-line set-up corrections were performed using an electronic portal imaging device (351 patients, 92.1%) or an on-board cone-beam CT (30 patients, 7.9%), as previously described (22).

**Table 1:** Patients and treatment characteristics and results of univariate analysis on acute toxicity.

		n° of pts (%)	Gastrointestinal				Genitourinary			
			Grade ≥ 2 (%)	χ (Fisher’s exact test)	Univariate logistic regression		Grade ≥ 2 (%)	χ (Fisher’s exact test)	Univariate logistic regression	
				p-value	OR	p-value		p-value	OR	p-value
Age	≤ 65	174 (45.7)	23.5	**0.032**	ref.		20.1	0.424	ref.	
	> 65	207 (54.3)	14.5		0.55	**0.024**	16.4		0.78	0.352
	CV	381 (100)			0.97	0.078			0.96	**0.044**
Charlson’s comorbidity index	0	309 (81.1)	19.4	0.161	ref.		18.1	0.971	ref.	
	1	57 (15.0)	17.5		0.88	0.741	19.3		1.08	0.833
	2	13 (3.4)	0		0.00	0.982	15.4		0.82	0.802
	3	2 (0.5)	50.0		4.15	0.317	0		0.00	0.983
Age adjusted Charlson’s comorbidity index	0	6 (1.6)	16.7	0.173	ref.		33.3	0.237	ref.	
	1	49 (12.9)	16.3		0.98	0.983	15.7		0.88	0.892
	2	178 (46.7)	24.7		1.64	0.655	16.8		0.37	0.268
	3	119 (31.2)	12.6		0.72	0.772	16.0		0.40	0.314
	4	25 (6.6)	12.0		0.68	0.761	0		0.38	0.346
	5	3 (0.8)	0		0.00	0.987	0		0.0	0.986
	6	1 (0.3)	0		0.00	0.992	0		0.0	0.992
PNI	No	84 (22)	13.1	0.187	ref.		10.7	0.053	ref.	
	Yes	297 (78)	20.2		1.68	0.143	20.2		2.11	0.050
Hypofractionation	No	127 (33.3)	15.7	0.376	ref.		15.7	0.480	ref.	
	Yes	254 (66.7)	20.1		1.34	0.307	19.3		1.28	0.398
Lymphadenectomy	No	94 (24.7)	21.3	0.288	ref.		19.1	0.706	ref.	
	< 15*	121 (31.8)	14.0		0.60	0.166	15.7		0.79	0.507
	≥ 15*	166 (43.8)	20.5		0.95	0.879	1.2		1.01	0.980
EQD_2_ prostate bed α/β_10_ (Gy)	≤ 68.3	193 (50.7)	17.3	0.699	ref.		16.2	0.358	ref.	
	> 68.3	188 (49.3)	19.7		1.15	0.605	20.2		1.32	0.294
	CV	381 (100)			1.00	0.164			1.00	0.103
Radiotherapy Technique	3D-CRT	94 (24.7)	13.8	0.214	ref.		13.8	0.271	ref.	
	IMRT	273 (71.7)	20.9		1.64	0.136	20.1		1.57	0.177
	VMAT	14 (3.7)	7.1		0.48	0.496	7.1		0.48	0.496
Image guidance	EPID	351 (92.1)	18.8	1	ref.		18.8	0.480	ref.	
	CB-CT	30 (7.9)	16.7		0.86	0.773	10.0		0.48	0.239
ADT	No	127 (33.)	18.9	1	ref.		13.4	0.120	ref.	
	Yes	254 (66.7)	18.5		0.97	0.926	20.5		1.67	0.092
Type of ADT	None	127 (33.3)	18.9	0.381	ref.		13.4	0.108	ref.	
	LHRH	183 (48.0)	16.4		0.84	0.568	18.6		1.48	0.227
	HD-Bic	71 (18.6)	23.9		1.35	0.402	25.4		2.20	**0.036**
EQD_2_ lymph nodes α/β_10_ (Gy)	≤ 44.3	280 (73.5)	18.2	0.839	ref.		16.4	0.204	ref.	
	> 44.3	101 (26.5)	19.8		1.11	0.725	22.8		1.5	0.158
	CV	381 (100)			1.00	0.126			1.0001	**0.035**

ADT, adjuvant deprivation therapy; CV, Continuous variable; PNI, prophylactic nodal irradiation.

Bold values means p-value less than 0.05.

### Acute and late toxicity

**Table 1** provides the results in terms of acute toxicity. None of the patients experienced acute toxicity greater than Grade 3, and the rates of Grade 3 gastrointestinal (GI) and genitourinary (GU) toxicity were 0.5% and 1.3%, respectively. The actuarial 5-year rates of Grade ≥ 2 GI and GU late toxicity-free survival were 90.4% and 83.5%, respectively. The actuarial 5-year rates of Grade ≥ 3 GI and GU late toxicity-free survival were 98.1% and 94.5%, respectively.

### Univariate analysis

Univariate analysis revealed that acute Grade ≥ 3 GI and GU toxicity rates were not significantly correlated with any of the analyzed parameters. However, the delivery of PNI showed a trend for correlation with higher rates of Grade ≥ 2 acute GU toxicity (**Table 1**).

The actuarial 5-year late Grade ≥ 2 GI toxicity was significantly lower in patients treated with hypofractionation (dose per fraction > 2 Gy compared to ≤ 2 Gy; 93.6% vs 84.0%; p: 0.006), IMRT or VMAT techniques (compared to 3D-conformal therapy; 93.2-100.0% vs 82.6%; p: 0.027), and PNI (compared to irradiation of the prostate bed only; 92.9% vs 80.2%; p: 0.009). Moreover, actuarial 5-year late Grade ≥ 2 GU toxicity did not show any significant correlation with the analyzed parameters. Furthermore, actuarial 5-year late Grade ≥ 3 GI toxicity was significantly lower in patients treated with hypofractionation (dose per fraction > 2 Gy compared to ≤ 2 Gy; 99.2% vs 96.1%; p-value: 0.033) and IMRT or VMAT techniques (compared to 3D-conformal therapy; 100.0% vs 93.5%; p-value: 0.022). Late Grade ≥ 3 GU toxicity did not exhibit any significant correlation with the analyzed parameters (**Table 2**).

**Table 2:** Actuarial 5-year gastrointestinal and genitourinary late toxicity-free survival rates (Grade ≥ 2 and Grade ≥ 3; Kaplan-Meier) and results of univariate analysis (log-rank).

		No of pts (%)	Gastrointestinal				Genitourinary			
			G ≥ 2 (%)	P	G ≥ 3 (%)	p	G ≥ 2 (%)	P	G ≥ 3 (%)	P
Age	≤ 65 years	174 (45.7)	94.1	.065	100.0	**.037**	83.4	.909	94.3	.683
	> 65 years	207 (54.3)	87.2		96.5		83.7		94.6	
Charlson’s comorbidity Index	0	309 (81.1)	91.5	.331	98.1	.900	82.6	.890	93.9	.688
	1	57 (15.0)	85.6		98.1		84.4		98.2	
	2	13 (3.4)	83.9		100.0		92.3		92.3	
	3	2 (0.5)	100.0		100.0		100.0		100	
Age adjusted Charlson’s comorbidity index	0	6 (1.6)	100.0	.396	100.0	.811	75.0	.865	100.0	,651
	1	49 (12.9)	95.8		100.0		90.9		96.8	
	2	178 (46.7)	92.5		98.3		79.8		93.2	
	3	119 (31.2)	85.4		97.4		85.4		95.8	
	4	25 (6.6)	83.0		95.7		86.6		92.0	
	5	3 (0.8)	100.0		100.0		64.9		100.0	
	6	1 (0.3)	100.0		100.0		100.0		100.0	
Nodal irradiation	No	84 (22.0)	80.2	**.009**	96.8	.288	87.7	.139	98.0	.204
	Yes	297 (78.0)	92.9		98.4		82.4		93.5	
Hypofractionation	No	127 (33.3)	84.0	**.006**	96.1	**.033**	83.2	.764	93.2	.533
	Yes	254 (66.7)	93.6		99.2		83.6		95.2	
Lymphadenectomy	No	94 (24.7)	92.6	.098	98.6	.259	79.2	.464	95.9	.758
	< 15*	166 (43.6)	92.9		99.2		86.9		94.1	
	≥ 15*	121 (31.8)	84.4		96.0		83.0		93.9	
EQD_2_ to the prostate bed α/β_3_ (Gy)	≤ 68.3	226 (59.3)	91.1	.808	98.1	.973	84.2	.603	92.7	.120
	> 68.3	155 (40.7)	89.3		98.2		83.2		97.0	
Radiotherapy technique	3DCRT	94 (24.7)	82.6	**.027**	93.5	**.002**	83.4	.524	96.4	.692
	IMRT	273 (71.7)	93.2		100.0		83.1		93.2	
	VMAT	14 (3.7)	100.0		100.0		100.0		100.0	
Image guidance	EPID	351 (92.1)	90.0	.455	98.0	.556	82.4	.059	94.1	.319
	CB	30 (7.9)	96.6		100.0		100.0		100.0	
Previous abdominal or pelvic surgery	No	367 (96.3)	90.3	.914	98.1	.662	83.6	.718	94.3	.467
	Yes	14 (3.7)	90.0		100.0		84.4		100.0	
Adjuvant Hormone Therapy	No	127 (33.3)	89.2	.836	98.0	.829	85.9	.341	95.6	.267
	Yes	254 (66.3)	91.0		98.2		82.3		93.9	
EQD_2_ to the lymph node α/β_3_ (Gy)	No	84 (22.0)	80.2	**.033**	96.8	.329	87.7	.236	98.0	.121
	≤ 43.2	196 (51.4)	93.0		99.5		82.2		91.7	
	> 43.2	101 (26.5)	92.7		96.6		83.5		96.9	
Acute GI toxicity	G 0	162 (42.5)	91.3	.601	99.0	.442	NA	NA	NA	NA
	G 1	148 (38.8)	88.2		97.1		NA	NA	NA	NA
	G 2-3	71 (18.6)	92.5		98.3		NA	NA	NA	NA
Acute GU toxicity	G 0	150 (39.4)	NA	NA	NA	NA	90.7	**.000**	95.3	.390
	G 1	162 (42.5)	NA	NA	NA	NA	85.5		92.1	
	G 2-3	69 (18.1)	NA	NA	NA	NA	65.2		98.0	

3D-CRT, 3-dimensional conformal radiotherapy, CB, cone beam; EQD_2_, Equivalent Dose in 2 Gy/fraction; EPID, Electronic portal imaging device; G, Grade; GI, gastrointestinal; GU, genitourinary; NA, not assessed; No, Number; Pts, patients; *number of resected lymph nodes.

Bold values means p<0.1.

### Multivariate analysis

The multivariable analysis of acute toxicity, conducted using binomial logistic regression, revealed a statistically significant association between older age and a reduced risk of Grade ≥ 2 GI acute toxicity (age analyzed as a dichotomous variable: OR: 0.569; 95% confidence interval [_95%_CI]: 0.329-0.973; p: 0.040). Apart from age, no other variable fitted in the multivariable logistic model for GI Grade ≥ 2 toxicity. Moreover, older age was significantly associated to a lower risk of Grade ≥ 2 GU acute toxicity (age analyzed as a continuous variable: OR: 0.956; _95%_CI: 0.918-0.996; p: 0.031). Regarding dichotomous variable GU Grade ≥ 2 acute toxicity, three variables were included in the multivariable model, including age as a continuous variable, ADT, and EQD2 α/β=10 to the prostate bed. While ADT and EQD2 enhanced the predictive model, they were not statistically significant (ADT: OR: 1.730, _95%_CI: 0.966-3.234, p: 0.073; EQD2: OR: 1.0005, _95%_CI: 0.9999-1.0010, p = 0.075). In contrast, the age variable remained statistically significant, with age showing an inverse association with toxicity (OR: 0.956, 95% CI: 0.918-0.996, p: 0.031). The multivariable analysis of late toxicity confirmed only a lower risk of Grade ≥ 2 GI toxicity in patients undergoing hypofractionation (OR: 0.38; _95%_CI: 0.18-0.78; p: 0.008). (**Table 3**).

**Table 3:** Multivariable analysis of late gastrointestinal and genitourinary toxicity.

Gastrointestinal toxicity (Grade ≥ 2)				
*Variable*	*Value*	*Hazard Ratio*	*95%CI*	*p=*
Age	CV	1.036	0.976-1.107	0.232
Charlson’s comorbidity index	0	Ref		
	> 0	1.678	0.953-2.955	0.073
Nodal irradiation	No	Ref		0.802
	Yes	0.974	0.593-1.498	
Hypofractionation	No	Ref		0.008
	Yes	0.381	0.184-0.783	
Lymphadenectomy	No/sampling	Ref		0.802
	Yes (>15)	0.942	0.589-1.505	
EQD_2_ to the prostate bed α/β_3_ (Gy)	CV	1.000	0.998-1.002	0.936
Radiotherapy technique	3D-CRT	Ref		0.929
	IMRT/VMAT	0.945	0.275-3.251	
Image guidance	EPID	Ref		0.267
	Cone-beam CT	0.561	0.202-1.557	
EQD2 to the lymph node α/β_3_ (Gy)	CV	1.001	0.998-1.003	0.580
Genitourinary toxicity (Grade ≥ 2)				
*Variable*	*Value*	*Hazard Ratio*	*95%CI*	*p=*
Age	CV	0.991	0.945-1.040	0.710
Charlson’s comorbidity index	0	Ref		0.468
	> 0	0.807	0.452-1.440	
Nodal irradiation	No	Ref		0.890
	Yes	0.744	0.110-4.894	
Hypofractionation	No	Ref		0.250
	Yes	0.544	0.193-1.534	
Lymphadenectomy	No/sampling	Ref		0.074
	Yes (>15*)	0.724	0.508-1.031	
EQD_2_ to the prostate bed α/β_3_ (Gy)	CV	1.001	0.998-1.003	0.120
Radiotherapy technique	3D-CRT	Ref		0.621
	IMRT/VMAT	0.767	0.269-2.191	
Image guidance	EPID	Ref		0.400
	Cone-beam CT	0.803	0.606-1.365	
EQD2 to the lymph node α/β_3_ (Gy)	CV	1.000	0.999-1.001	0.972
Acute GU toxicity	Grade 0-1	Ref		0.059
	Grade 2-3	3.060	0.882-13.658	

3D-CRT,3-dimensional conformal radiotherapy; EQD2, Equivalent Dose in 2 Gy/fraction; EPID, Electronic portal imaging device; *number of resected lymph nodes.

## Discussion

Adjuvant RT has been associated with an increased risk of side effects compared to surgery alone (4) and early salvage RT (7–9). However, it is important to note that, in selected high-risk PCa patients, adjuvant RT offers a higher chance of cure compared to surgery alone. Our multicenter observational study confirms that severe acute toxicity is rare in this setting. The rates of acute Grade ≥ 3 GI and GU toxicity were only 0.5% and 1.3%, respectively, and the 5-year actuarial cumulative incidence of late Grade ≥ 3 GI and GU toxicity rates were 1.9% and 5.5%, respectively.

Furthermore, our analysis demonstrated lower rates of GI acute toxicity in older patients. This unexpected result may arise from the fact that elderly patients may be more likely to have pre-existing symptoms or discomfort due to age-related health issues or comorbidities. As a result, they might be less inclined to report or attribute certain side effects to RT, especially if these side effects are mild or non-serious. The phenomenon of underreporting or downplaying side effects in elderly patients is known as “response shift” or “response shift bias” (23).

Other studies have reported an increased risk of GI early adverse effects in patients with higher mean rectal dose (16) or larger irradiated bowel volumes (24), those receiving PNI (25, 26), individuals with previous abdominal surgery (24), and those under anticoagulant or antiplatelet therapy (16). Additionally, Fiorino et al. observed reduced toxicity rates in patients receiving IMRT (24), although this effect was not observed in our cohort or in the study by Flores-Balcazar et al. (27).

Furthermore, our study demonstrated a reduced risk of GU acute toxicity in older patients, while Martinez-Arribas et al. reported higher GU acute toxicity rates in patients with urinary symptoms before RT. Additionally, similar to our findings, Flores-Balcazar et al. (27) and Deville et al. (26) did not observe a significant impact of IMRT/VMAT and PNI, respectively.

Moreover, our analysis revealed a reduced risk of GI late toxicity in patients treated with hypofractionated RT. Another study observed a higher risk of late GI adverse effects in subjects with higher body mass index values and those treated with higher RT doses (17). Furthermore, Flores-Balcazar et al. did not find a significant impact of IMRT/VMAT, in line with our findings, while Goenka et al. reported significantly reduced toxicity in patients treated with IMRT (28). Similarly, Deville et al. did not find different toxicity rates in subjects treated with PNI (26). **.

In our analysis, no parameter was significantly correlated with late GU toxicity. However, other studies have reported a significant correlation between higher toxicity rates and older age and receiving > 70 Gy to larger bladder volumes (17), hypofractionated RT (15), and Grade > 2 acute GU toxicity (13, 15). Interestingly, IMRT did not show an impact on late GU toxicity in two studies (27, 28), consistent with our analysis. Waldstein et al. reported increased toxicity rates in patients treated with PNI (25), while Deville et al. did not observe this correlation (26), similar to our series.

In conclusion, the results of available evidence conflict regarding: i) the impact of modulated RT techniques on acute GU toxicity and late GI side effects, and ii) the impact of PNI on late GU toxicity. Moreover, there is limited evidence available regarding parameters predicting acute GU side effects.

The use of hypofractionation in the adjuvant RT setting of PCa remains a controversial topic. Moderately hypofractionated regimens are considered preferable in patients undergoing exclusive RT (NCCN 2022) but not in the adjuvant setting. According to the NCCN guidelines, the recommended standard fractionation dose for adjuvant/salvage RT is 64-72 Gy (NCCN 2022). However, the data available on this topic are very heterogeneous. For instance, a systematic review on hypofractionated postoperative RT reported rates of Grade ≥ 2 late GU toxicity ranging between 0% and 66% (29).

The results of our analysis did not indicate a worse toxicity profile in patients undergoing hypofractionated RT. Furthermore, the multivariable analysis revealed a reduced rate of late GI toxicity after RT delivered with > 2 Gy per fraction. In contrast, Cozzarini et al. reported a significant increase in the rate of Grade ≥ 3 GU toxicity in patients receiving hypofractionated regimens compared to conventional fractionation (5-year risk: 18.1% versus 6.9%). This difference can be explained by comparing the equivalent doses delivered in our study and Cozzarini’s et al. study. Assuming an α/β ratio of 3 Gy for late effects, patients undergoing hypofractionation in our study received a median dose of 68.7 Gy, while in Cozzarini’s et al. study, the range was 68.4-80.8 Gy. Moreover, in Cozzarini’s et al. study, the EQD2 was > 70 Gy in 79.8% of patients and > 79 Gy in 32.4% of subjects. Additionally, the EQD2 for PNI was 43.2 Gy in our series and 50.2 Gy in Cozzarini’s et al. series. Even when using an α/β ratio of 5, as done by Cozzarini et al., our median EQD2 (67.0 Gy) was lower compared to their analysis (median: 70.4 Gy, IQR: 70.4-79.2 Gy).

Taken together, the results from the two studies suggest a possible association between dose and late urological toxicity in this setting, highlighting the need for further investigation. It is also worth noting that the safety of hypofractionation observed in our data is consistent with recent analyses (30–32). Probably, the lower incidence of late toxicity recorded in patients treated with hypofractionation in our study, despite a significantly higher EQD2_α/β=3_ value, may derive from the delivery of RT in more recent times, and therefore with more precise techniques.

The paradoxical result of our analysis, of reduced late gastrointestinal toxicity in patients undergoing PNI, remains to be explained. The only interpretation we can propose is that patients with better general conditions and fewer comorbidities (particularly at the intestinal level) were more frequently referred to PNI.

Our study has certain limitations. The scales used to score acute and late toxicity are outdated, and an assessment of the treatment impact on quality of life is lacking. Furthermore, despite efforts to include as many parameters as possible in the analysis, some were missing from our database. Among these, several factors have shown a significant impact on toxicity rates in previous studies, such as baseline symptoms (16) drug therapy during RT (16), planning dose/volume indices (14, 17), body mass index (17), and tobacco history (17).

On the other hand, the strengths of this study lie in the large number of cases analyzed and the comprehensive inclusion of numerous parameters related to both patients and treatments, as well as RT techniques in the analysis.

In conclusion, the results of our analysis demonstrate that although adjuvant RT significantly increases the overall rate of adverse events in PCa patients, the risk of severe toxicity is low. Additionally, acute toxicity rates were higher in younger patients, while a protective effect of hypofractionation was observed in terms of late GI toxicity.

To minimize the negative impact of adjuvant RT, further studies are warranted. These analyses should aim to: i) develop predictive models of toxicity combined with the risk of recurrence based on a comprehensive range of clinical, genetic-molecular, and treatment-related parameters, to guide the careful selection of patients for immediate adjuvant RT; ii) analyze toxicity rates in patients undergoing tailored/intensified adjuvant RT. For example, studies have shown that biochemical relapse-free survival can be improved by modulating postoperative RT, such as adjusting the dose based on surgical margin status, delivering PNI in selected cases, and administering ADT based on the risk of treatment failure (33–36); iii) clarify the impact of hypofractionation on late GU toxicity, given the conflicting evidence in the literature (29).

## Data availability statement

The raw data supporting the conclusions of this article will be made available by the authors, without undue reservation.

## Ethics statement

The studies involving humans were approved by Ethics Committee of IRCCS Azienda Ospedaliero-Universitaria di Bologna (311/2019/Oss/AOUBo, ICAROS-1 study). The studies were conducted in accordance with the local legislation and institutional requirements. The participants provided their written informed consent to participate in this study.

## Author contributions

MB: Conceptualization, Formal analysis, Writing – original draft, Writing – review & editing. GM: Conceptualization, Writing – review & editing. LC: Data curation, Investigation, Writing – review & editing. AC: Data curation, Investigation, Writing – review & editing. CM: Formal analysis, Writing – review & editing. LB: Writing – review & editing. MN: Writing – review & editing. AA: Data curation, Formal analysis, Writing – review & editing. IC: Data curation, Investigation, Writing – review & editing. EN: Writing – review & editing. SCi: Formal analysis, Writing – review & editing. FC: Writing – review & editing. LT: Writing – review & editing. LS: Writing – review & editing. SCa: Data curation, Writing – review & editing. RS: Writing – review & editing. EB: Writing – review & editing. AGM: Conceptualization, Supervision, Writing – original draft, Writing – review & editing. FD: Conceptualization, Supervision, Writing – review & editing.

## Footnotes

1
https://uroweb.org/guidelines/prostate-cancer/chapter/treatment.

2
http://www.nccn.org/professionals/physician_gls/pdf/prostate.pdf.
